# The influence and impact of smoking on red blood cell morphology and buccal microflora: A case‐control study

**DOI:** 10.1002/jcla.23212

**Published:** 2020-01-16

**Authors:** Khalid Hadi Aldosari, Gulfam Ahmad, Sameer Al‐Ghamdi, Mohammed H. Karrar Alsharif, Abubaker Y. Elamin, Muhammad Musthafa, Mohammed Y. Abbas, Abdulaziz Abdullah Alqarni, Suliman Khaled Alqudeebi, Abdullah Ali Binsaqer, Hussain Al‐Ghamdi

**Affiliations:** ^1^ Colleges of Medicine Prince Sattam Bin Abdulaziz University Al Kharj Saudi Arabia; ^2^ Human Reproduction Unit Kolling Institute Sydney Medical School Sydney University Sydney NSW Australia; ^3^ Departemnt of Family Medicine College of Medicine Prince Sattam bim Andulaziz University Al Kharj Saudi Arabia; ^4^ Department of Basic Medical Sciences Colleges of Medicine Prince Sattam Bin Abdulaziz University Al Kharj Saudi Arabia; ^5^ Department of Histology & Embryology Medical Faculty Ondokuz Mayis University Samsun Turkey; ^6^ Department of Anatomy Faculty of Medicine National University Khartoum Sudan; ^7^ Emergency Medical Specialties Department Al‐Ghad International Colleges for Applied Medical Sciences Al Madinah Al Munawarah Saudi Arabia; ^8^ Microbiology Department Colleges of Medicine Prince Sattam Bin Abdulaziz University Al Kharj Saudi Arabia; ^9^ Medical Laboratory Sciences Allied Health Department College of Health Sciences University of Bahrain Manama Kingdom of Bahrain; ^10^ Department of Infection Control Al‐Thager General Hospital Jeddah Saudi Arabia

**Keywords:** microflora, morphology, RBCs, smokers

## Abstract

**Background:**

Tobacco smoking is a major health issue worldwide. In addition to several health problems, smoking can also cause buccal cavity ulcers and buccal cavity cancer in case of chronic smoking. Tobacco smoking may also lead to deranged morphology of red blood cells (RBCs), which results in reduced oxygen carrying capacity of the blood.

**Aim:**

(a) To investigate and compare the changes in the RBC morphology of smokers and nonsmokers. (b) To investigate and compare the normal buccal flora of smokers and nonsmokers.

**Methodology:**

A total of 81 men were included in the study. Study population was divided into two groups: group 1; smokers (n = 50) and group 2; nonsmokers, which served as control (n = 31). After informed written consent from the study participants, a 5 mL of venous blood was drawn under sterile conditions for complete blood analysis and RBC morphology. Samples from buccal cavity were collected by cotton swab and cultured in sterile petri dishes to identify the bacterial growth. Data of RBC morphology and buccal microbiota were compared between smokers and nonsmokers.

**Results:**

Buccal microflora results showed heavy growth in smokers compared with nonsmokers. Mean values of RBCs, Platelets, WBCs, HGB (hemoglobin), and MCV (mean corpuscular volume) did not differ between smokers and nonsmokers. Mean red cell distribution (RDW) width significantly was lower in smokers than nonsmokers. Macrocytic RBCs was more in smokers (60%) compared with nonsmokers (4%).

**Conclusions:**

Our results showed an increase in the percentage of macrocytic RBCs and a decrease in the red cell distribution width (RDW) in smokers compared with nonsmokers. Buccal Microflora was significantly higher in smoker group in contrast to nonsmoker group.

## 1 BACKGROUND

Tobacco smoking is one of the most serious problems that the public health faces all over the world. It has been estimated that the number of tobacco smokers has increased from 721 million in 1980 to 976 million in 2012.^1^ According to American Cancer Society, “tobacco smoking kills Americans more than alcohol, car accidents, HIV and illegal drugs combined.” Moreover, tobacco smoking is a leading cause for many serious diseases and it could cause cancer. A relation between tobacco smoking and cancer of lung, oral cavity, larynx, pharynx and pancreas has been reported.^2^ Tobacco smoking is not only about morbidity but also leads to mortality. According to American Cancer Society, 30% of the cancer deaths in the United States are because of tobacco smoking, which includes 80% of deaths from the lung cancer.^3^ Furthermore, the risk of smoking is not limited to the active tobacco smokers only. According to World Health Organization (WHO), the number of deaths because of smoking is 7 million each year, of which 6 million are active smokers while around 0.9 million are passive smokers.

Cigarette smoke contains around 5000 chemicals including carbon monoxide (CO) and tars. Many of these substances are considered toxic for human body.^4^, ^5^ In alveolar capillaries, CO can diffuse rapidly and binds to hemoglobin (Hb) firmly (with binding ability of 200‐250 times greater than that of O2) leading to tissue hypoxia because of the formation of carboxyhemoglobin (HbCO) and leading to elevated values of Hb and red blood cells (RBCs).^6^

Tobacco smokers may have increased level of HB and hematocrit. A direct correlation between RBC count and number of cigarettes smoked per day has been reported.^7^ Additionally, tobacco smoking shows harmful effects on white blood cells (WBCs) in smokers of both genders. It has been reported that smokers of both genders have significantly higher number of leukocytes and elevation in neutrophils, lymphocytes, leukocytes, monocytes, eosinophils, and basophils compared with nonsmokers.^8^

Morphological changes in complete blood count (CBC) show change in mean corpuscular volume (MCV) because of the effects of CO on erythrocytes.^9^ This occurs because of CO hypoxia, which produces a demand for more erythrocytes. Because of cyanide‐induced diversion of vitamin B12, the demand cannot be met, which leads to change in RBC morphology.^10^

The human oral cavity is composed of different structures like tongue, teeth, and soft and hard palate, which are inhabited by numerous microorganisms.^11^ For example, the oral cavity is colonized by more than 400 bacterial species, named as the oral commensal. These organisms are beneficial for human health and participate in maintaining oral health. If dysbiosis occurs, it may result in pathological conditions, such as gingivitis, in more severe cases neck cancer. Oral dysbiosis may increase the risk of pulmonary, heart, and digestive system diseases.^12^ The dysbiosis may result because of smoking as tobacco may affect the commensal of oral cavity due to its direct contact with oral mucosa.^13^ In this context, the goal of the present study is to investigate the RBC morphological changes and changes in the oral microbiota of the active smokers.

Cigarette smoking is one of the leading risk factors of many grave diseases such as cardiovascular problems and lung, esophageal, and gastrointestinal cancers. The present study aims to highlight the relationship between cigarette smoking and morphological changes of red blood cells (RBCs) as well as its effect on oral microbiota. RBCs are the oxygen‐carrying cells and are essential for normal body working especially under stress where more oxygen is required. Any change in the morphology of RBCs may lead to reduced oxygen carrying capacity, which can significantly compromise the body functions. On the other hand, smoking can kill the normal microflora of the oral cavity, which maintains the healthy oral environment. The normal buccal flora makes a biofilm on the surface of normal dentation, which can be compromised in smokers. The present study will compare the RBC morphology and microflora between smokers and nonsmokers.

## 2 METHODS

A case‐control study was performed with the attendee of primary care clinics of family and community medicine department in a secondary care hospital in Al‐Kharj City, Saudi Arabia, from September 2017 to the end of March 2018. The study was conducted by enrolling patients who made unscheduled visits to the primary care clinics. After informed consent, a total of 81 men, with age range of 18‐50 years, were included in the study. Study population was divided into two groups: cases (smokers) and control (nonsmokers).

For data collection, two trained physicians interviewed participants using an 8‐item Arabic questionnaire. The questionnaire used was based on a review of the published literature. The following information was collected: part 1: demographic data, part 2: smoking status and habit, and part 3: current medical history.

### 2.1 Inclusion and exclusion criteria

Selection criteria for smokers included men who had been smoking for at least 1 year. However, those who have started smoking for a month were also recruited to compare with chronic smokers. Smokers having any other associated disease such as cardiac, lung, or stomach disease were excluded. Men who never smoked and had never been diagnosed with cardiac and lung diseases were included in control group.

### 2.2 Blood test

Under sterile and aseptic conditions, 5 mL venous blood was drawn from each participant in EDTA vial. Blood sample was drawn in the laboratory to avoid any transportation mishandling. A complete blood count analysis was performed to obtain the complete blood picture. RBCs morphology was assessed by automated machine. Although smoking alters various hematological parameters including hemoglobin, hematocrit, and leukocytes; however, the study was limited to assess the morphology of RBCs in detail. In addition, effect of oxygen is more prominent on the RBC membrane as compared to any other tissue of the body.

### 2.3 Oral cavity swab

Oral cavity samples were collected by cotton swab by an expert physician. Swabs were sent to the laboratory for the culture. After 24 hours of culture sensitivity, the microflora were identified in both the groups. Oral cavity swabs were used in the study as tobacco smoking is one of the potential environmental factors, which affects buccal flora directly, as well as, indirectly in terms of oxygen deprivation, immune modulation, oral biofilm formation, and several other pathological mechanisms. In addition, it is simple and convenient to collect oral cavity swabs.

### 2.4 Data analysis

All data were collected and analyzed to compare the normal blood profile in individual patients, and means of RBCs, Platelets, WBCs, HGB (hemoglobin), MCV (mean corpuscular volume), and RDW (red cell distribution width) were compared between the two groups by paired *t* test. RBC morphology was analyzed in some of the patients, and the abnormality was identified. Buccal microflora population was analyzed and compared between smokers and nonsmokers.

### 2.5 Ethical approval

The local institutional review board approved the study protocol. Confidentiality of the gathered information of the participants’ and clinical data had been assured, and a written informed consent from each respondent was gathered as a personal permission to take part in the study.

## 3 RESULTS

Mean age of smokers and nonsmokers was 25.8 ± 5.9 (range of 20‐42 years) and 22.6 ± 1.8 (range of 20‐25 years), respectively. All participants were of Arab origin. Average time since smoking was 7.54 years with a range of 1‐20 years. Comparison of weekly physical activity and vitamin supplementation and RBCs morphological abnormality is given in Table 1.

**Table 1:** Comparison of physical activity, supplements, and RBCs morphology between smokers and nonsmokers

Parameters	Populations	
	Smokers	Nonsmokers
Weekly exercise	4	8
Shortness of breath	40	4
VitB12 deficiency	8	0
VitB12 supplements	4	0
Macrocytic RBCs	60	4

**Figure 1:**
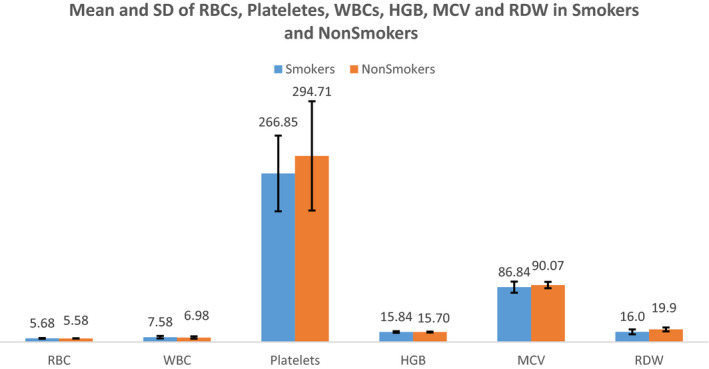
Comparison of values of RBCs, Platelets, WBCs, HGB, MCV, and RDW between smokers and nonsmokers

Values of RBCs, Platelets, WBCs, HGB (hemoglobin), MCV (mean corpuscular volume), and RDW (red cell distribution width) of the smokers and nonsmokers are given in Table 2 and Figure 1, respectively. Mean values were compared between smokers and nonsmokers and are presented in Table 2. Red cell distribution width (RDW) was significantly lower in smokers compared with nonsmokers.

**Table 2:** Values of RBCs, Platelets, WBCs, HGB (hemoglobin), MCV (mean corpuscular volume), and RDW (red cell distribution width) of smokers and nonsmokers

Outcome	Group						95% CI for Mean Difference	*t* test results		
	Smokers			NonSmokers						
	M	SD	n	M	SD	n		*t*	*df*	Sig. (2‐tailed)
RBC	5.6817	0.55887	46	5.5832	0.22029	31	−0.0841, 0.281	1.078	63.110	0.285
WBC	7.5783	2.02576	46	6.9765	1.79378	31	−0.295, 1.498	1.338	75	0.185
Platelets	266.8478	59.98443	46	294.7097	86.56720	31	−61.103, 5.379	−1.670	75	0.099
HGB	15.8435	1.31684	46	15.7032	0.72041	31	−0.3248, 0.6053	0.601	72.420	0.550
MCV	86.8391	8.80892	46	90.0710	5.01233	31	−6.381, −0.0825	−2.045	73.261	0.044*
RDW	16.0326	3.89364	46	19.8855	3.10562	31	−5.519, −2.187	−4.607	75	0.000**

* ≤ 0.05.

** ≤ 0.001.

**Figure 2:**
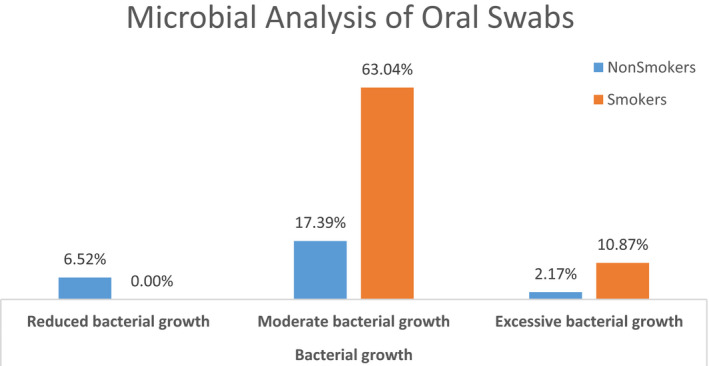
Microbial analysis of oral swabs of smokers and nonsmokers

Buccal microflora results showed heavy growth in smokers compared with nonsmokers. 6.52% of non‐smoker showed reduced bacterial growth compare with 0% in the smoker group. Moderate bacterial growth was significantly higher in smoker group with 63.04% compared with nonsmoker group with 17.30%. Excessive bacterial growth was higher in smoker group with 10.87% in contrast to 2.17% in nonsmoker group. However, due to logistics and financial constraints we could not identify the individual species of microbes between smokers and nonsmokers. Comparison is given in Figure 2.

## 4 DISCUSSION

Despite the fact that smoking is injurious to health, the trend of smoking is high throughout the globe. A large population starts smoking as recreational activity, and then, it turns into an addiction. We planned this study to compare the blood parameters of smokers with nonsmokers. The major focus was on the red blood cell morphology because these are oxygen‐carrying cells. Any abnormality in the number or synthesis of RBCs may lead to reduced oxygen carrying capacity ultimately causing difficulties in daily life activities. In our study population, the average time to smoking was 7.54 years and 40% of the smokers complained of shortness of breath. Sixty percent of smokers showed macrocytic RBCs compared with 4% nonsmokers. This reflects that smoking has an underlined linkage, which affects the morphology of the RBCs. Surprisingly, the red cell distribution width (RDW) was lower in smokers than nonsmokers. This finding is contradictory to what we found in overall morphology of RBCs. The actual reason for this discrepancy is still to rule out but we speculate that this might be because of different machines used for the analysis, which might have affected the results.

The second objective of the study was to identify the buccal flora in smokers and nonsmokers. The buccal swabs of the smokers and nonsmokers revealed growth of normal microflora. However, the swab cultures of smokers showed heavy growth compared with nonsmokers. Unfortunately, we were unable to identify the individual species if microbes in smokers and nonsmokers, which could have provided us with more precise results about the difference, if any, in microflora population of smokers and nonsmokers.

### 4.1 Limitations

We encountered few limitations during the study period that affected the expected outcome of the study. These include the shortage of time to complete the study and lack of cooperation and unavailability of research funds at University Hospital. The collaboration with military hospital took extra time, which caused some delay in the sample collection and analysis. Collection of sufficient number of samples was also difficult due to limited cooperation of patients.

## 5 CONCLUSIONS

Our results showed an increase in the percentage of macrocytic RBCs and a decrease in the red cell distribution width (RDW) in smokers compared with nonsmokers. Buccal microflora was significantly higher in smoker group in contrast to nonsmoker, which showed reduced bacterial growth in some samples.

## AUTHORS’ CONTRIBUTIONS

KHD and SAG designed the study. GA, SAG, and MH performed data acquisition. AYE, AAA, SKA, and AAB performed the analysis and drafted the manuscript. All authors read and approved the final manuscript for submission.

## ETHICS APPROVAL AND CONSENT TO PARTICIPATE

This study was ethically approved by the Institutional Review Board (IRB) of College of Medicine, Prince Sattam Bin Abdulaziz University. All study participants gave written informed consent as a personal permission to take part in the study.

## CONSENT FOR PUBLICATION

Not applicable.

## Data Availability

All data and materials are included within the manuscript and available upon request from the corresponding author.

## References

[1] Wakefield MA , Durkin S , Spittal MJ , et al. Impact of tobacco control policies and mass media campaigns on monthly adult smoking prevalence. Am J Public Health. 2008;98(8):1443‐1450. 18556601 10.2105/AJPH.2007.128991 PMC2446442

[2] Tobacco smoking. IARC Monogr Eval Carcinog Risk Chem Hum. 1986;38:35‐394. 3460963

[3] American Heart Association . Smoking & cardiovascular disease (heart disease). http://www.heart.org/idc/groups/heartpublic/@wcm/@adv/documents/downloadable/ucm_304555.pdf. Accessed February 17, 2014.

[4] Hoffmann D , Hoffmann I . Letters to the editor, tobacco smoke components. Beitr Tabaksforsch Int. 1998;18:49‐52.

[5] Borgerding M , Klus H . Analysis of complex mixtures—cigarette smoke. Exp Toxicol Pathol. 2005;57:43‐73. 16092717 10.1016/j.etp.2005.05.010

[6] Blumenthal I . Carbon monoxide poisoning. J R Soc Med. 2001;94:270‐272. 11387414 10.1177/014107680109400604 PMC1281520

[7] Lakshmi AS , Lakshmanan A , Kumar GP , Saravanan A . Effect of intensity of cigarette smoking on haematological and lipid parameters. J Clin Diagn Res. 2014;8(7):11‐13. 25177557 10.7860/JCDR/2014/9545.4612 PMC4149063

[8] YuEmail G , Phillips S , Gail MH , et al. The effect of cigarette smoking on the oral and nasal microbiota. Microbiome. 2017;5:3. 28095925 10.1186/s40168-016-0226-6 PMC5240432

[9] Asif M , Karim S , Umar Z , et al. Effect of cigarette smoking based on hematological parameters: comparison between male smokers and nonsmokers. Turk J Biochem. 2013;38(1):75‐80.

[10] Norman Helman MD , Rubenstein LS . The effects of age, sex, and smoking on erythrocytes and leukocytes. Am J Clin Pathol. 1975;63(1):35‐44. 1111274 10.1093/ajcp/63.3.35

[11] Dewhirst FE , Chen T , Izard J , et al. The human oral microbiome. J Bacteriol. 2010;192(19):5002‐5017. 20656903 10.1128/JB.00542-10 PMC2944498

[12] Jing WU , Peters BA , Dominianni C , et al. Cigarette smoking and the oral microbiome in a large study of American adults. ISME J. 2016;10:2435‐2446. 27015003 10.1038/ismej.2016.37 PMC5030690

[13] Macgregor ID . Effects of smoking on oral ecology. A review of the literature. Clin Prev Dent. 1989;11:3‐7. 2689047

